# The Dynamic Nature of Hypertrophic and Fibrotic Remodeling of the Fish Ventricle

**DOI:** 10.3389/fphys.2015.00427

**Published:** 2016-01-21

**Authors:** Adam N. Keen, Andrew J. Fenna, James C. McConnell, Michael J. Sherratt, Peter Gardner, Holly A. Shiels

**Affiliations:** ^1^Faculty of Life Sciences, University of ManchesterManchester, UK; ^2^Faculty of Medical and Human Sciences, Centre for Tissue Injury and Repair, University of ManchesterManchester, UK; ^3^School of Chemical Engineering and Analytical Science, Manchester Institute of Biotechnology, University of ManchesterManchester, UK

**Keywords:** compliance, fibrosis, heart, stiffness, temperature acclimation, phenotypic plasticity

## Abstract

Chronic pressure or volume overload can cause the vertebrate heart to remodel. The hearts of fish remodel in response to seasonal temperature change. Here we focus on the passive properties of the fish heart. Building upon our previous work on thermal-remodeling of the rainbow trout ventricle, we hypothesized that chronic cooling would initiate fibrotic cardiac remodeling, with increased myocardial stiffness, similar to that seen with pathological hypertrophy in mammals. We hypothesized that, in contrast to pathological hypertrophy in mammals, the remodeling response in fish would be plastic and the opposite response would occur following chronic warming. Rainbow trout held at 10°C (control group) were chronically (>8 weeks) exposed to cooling (5°C) or warming (18°C). Chronic cold induced hypertrophy in the highly trabeculated inner layer of the fish heart, with a 41% increase in myocyte bundle cross-sectional area, and an up-regulation of hypertrophic marker genes. Cold acclimation also increased collagen deposition by 1.7-fold and caused an up-regulation of collagen promoting genes. In contrast, chronic warming reduced myocyte bundle cross-sectional area, expression of hypertrophic markers and collagen deposition. Functionally, the cold-induced fibrosis and hypertrophy were associated with increased passive stiffness of the whole ventricle and with increased micromechanical stiffness of tissue sections. The opposite occurred with chronic warming. These findings suggest chronic cooling in the trout heart invokes a hypertrophic phenotype with increased cardiac stiffness and fibrosis that are associated with pathological hypertrophy in the mammalian heart. The loss of collagen and increased compliance following warming is particularly interesting as it suggests fibrosis may oscillate seasonally in the fish heart, revealing a more dynamic nature than the fibrosis associated with dysfunction in mammals.

## Introduction

Chronic changes in pressure or volume load can cause the vertebrate heart to change in size, form and function (Clark and Rodnick, 1999; Opie et al., 2006). The heart remodels in an attempt to ensure appropriate output, commonly achieved through hypertrophy which can be defined as an enlargement of a part or the whole of an organ due to an increase in the size of its constituent cells (Dorn, 2007). Hypertrophy of the mammalian left ventricle can improve cardiac performance to meet increased demands such as those occurring with pregnancy or following exercise training (Mone et al., 1996). This “physiological” hypertrophy increases ventricular wall thickness in line with chamber radius causing both stroke volume and systolic pressure to increase, improving overall cardiac output (Dorn, 2007; Bernardo et al., 2010). Importantly, under most conditions, this remodeling is transient and regresses when the stimulus is removed (Bernardo et al., 2010). Hypertrophy can also occur in response to chronic pathological stressors like hypertension. Here, ventricular wall thickness increases, but the luminal volume is reduced (Dorn, 2007) allowing systolic pressure to be maintained, but at the expense of stroke volume which can lead to both systolic and diastolic dysfunction. Pathological remodeling is persistent and is associated with various cardiomyopathies including myocardial infarction, arrhythmia, and sudden death (Bernardo et al., 2010).

The hearts of non-mammalian vertebrates also remodel to meet changing systemic demands. For example, the ventricular mass of the Burmese python, *Python molurus*, can increase by 40% after feeding and then return to “normal” following digestion (Andersen et al., 2005). The hearts of many fish also show intermittent remodeling in response to seasonal temperature change with chronic cooling during winter triggering ventricular hypertrophy (Farrell et al., 1988; Graham and Farrell, 1989; Tervonen et al., 2001; Klaiman et al., 2011; Shiels et al., 2011). The hypertrophic trigger in this model is thought to be the increased viscosity of blood at cold (<6°C) temperatures and the hemodynamic stress of pumping this viscous blood (Graham and Farrell, 1989; Clark and Rodnick, 1999). Importantly, the cold-induced hypertrophic phenotype is thought to regress during warming in summer (Klaiman et al., 2011). The temperature-induced cardiac remodeling in fish has, therefore, generally been considered analogous to the physiological cardiac remodeling that occurs in mammals following non-pathological hypertrophic stimuli.

Stimuli known to trigger pathological hypertrophic remodeling in mammals may also arise in fish following chronic cold. The acute effect of low temperature is to decrease contractile function (Aho and Vornanen, 1999), through direct (i.e., *Q*_10_; rate of reaction change over a 10°C temperature change) effects on the ion pumps and channels underlying cellular excitation-contraction coupling (Vornanen et al., 2002), and to reduce the Ca^2+^ sensitivity of contractile elements (Gillis et al., 2000) which must be compensated for. Furthermore, there is evidence for ventricular fibrosis in fish following chronic cold (Klaiman et al., 2011). In mammals, increased collagen fibril density can strengthen chamber walls and improve transduction of myocardial force (Collier et al., 2012). However, fibrosis can also cause excessive stiffness, which reduces diastolic and systolic function and increases the chance of arrhythmias (Chapman et al., 1990). Fibrosis is largely absent in mammalian physiological hypertrophy (Bernardo et al., 2010).

Pathological and physiological hypertrophy are also differentiated by markers of myocardial stretch, including atrial natriuretic peptide (ANP) and brain natriuretic peptide (BNP), and the activation of the signaling pathways that promote growth, such as the fetal gene program (de Bold and de Bold, 2005; Bernardo et al., 2010). Up-regulation of genes associated with mammalian pathological hypertrophy are also found in hypertrophic fish hearts following chronic cold or stress (Vornanen et al., 2005a; Johansen et al., 2011). Thus, thermal remodeling of the fish heart appears to exhibit characteristics of both physiological and pathological hypertrophy of the mammalian heart.

Here, using acclimation temperatures to simulate seasonal temperature change, we investigate the effects of chronic cooling (from 10 ± 1°C to 5 ± 1°C) and chronic warming (from 10 ± 1°C to 18 ± 1°C) on the rainbow trout ventricle to determine the extent of temperature-induced connective tissue remodeling and the passive properties of the ventricle across multiple levels of organization. This approach extends our previous study which described thermal remodeling of the active properties of the salmonid heart (Klaiman et al., 2011). We hypothesized that chronic cooling would increase myocardial stiffness, fibrosis and up-regulation of factors involved in pathological hypertrophy in mammals. To understand functional consequences of remodeling we used atomic force microscopy (AFM), to determine micromechanical ventricular stiffness, and generated *ex vivo* pressure-volume curves, to determine ventricular chamber compliance. As trout experience intermittent temperature change, we were particularly interested in variable remodeling following both warming and cooling. We found chronic cold induced a remodeling phenotype with aspects analogous to pathological hypertrophy in mammals, particularly relating to chamber stiffness and collagen deposition. The opposite response was found following chronic warming suggesting a reversible phenotype. The fish heart could thus provide a model to investigate the regression of fibrotic cardiac hypertrophy.

## Materials and methods

### Ethical approval

All husbandry and housing conditions were in accordance with the University of Manchester handling protocols and adhere to the UK Home Office legislation. All experimental procedures were approved by the University of Manchester ethical review committee.

### Experimental animals

Sexually mature female rainbow trout (*Onchorynchus mykiss*; *n* = 47; morphometric data in Table 1) were purchased from Dunsop Bridge Trout Farm (Clitheroe, UK), housed on a 12 h light: 12 h dark cycle in ~500 L re-circulated aerated fresh water tanks at 10 ± 1°C and fed to satiation 3 times per week. Water quality was ensured with 30% water changes 3 times per week and regular tests for temperature, pH, nitrates and nitrites. Fish were held under these conditions for a minimum of 2 weeks before being randomly assigned to one of three acclimation groups; cold (5 ± 1°C), control (i.e., no change; 10 ± 1°C), or warm (18 ± 1°C). These temperatures are based on previous literature which describes the cardiac remodeling response in salmonids (Klaiman et al., 2011). Water temperature of the warm and cold acclimation groups was changed by 1°C per day until desired temperature was reached and then held at that temperature for a minimum of 8 weeks before experimentation. The photoperiod for the cold acclimated animals was changed to 8 h light: 16 h dark cycle to simulate winter (Graham and Farrell, 1989).

**Table 1:** **The gross morphological parameters for cold acclimated (5°C), control (10°C) and warm acclimated (18°C) rainbow trout**.

	**Cold acclimated**	**Control**	**Warm acclimated**
Mass (g)	480.8±64.3	526.1±42.8	524.8±60.2
Heart mass (g)	0.94±0.09	1.15±0.12	0.99±0.11
RHM (g·mass^−1^)	0.0021±0.00010	0.0022±0.000086	0.0019±0.000079
Ventricular mass (g)	0.59±0.075	0.68±0.11	0.62±0.073
RVM (g·mass^−1^)	0.0013±0.000066	0.0012±0.00011	0.0012±0.000052

RHM, relative heart mass; RVM, relative ventricular mass. Values given are mean ± S. E. Significance was determined by GLM with Tukey post-hoc test for comparision between the groups (P < 0.05), n = 47 for each group.

### Tissue processing

Fish were killed by a blow to the head followed by severance of the spinal cord and destruction of the brain. The heart was excised, rinsed in phosphate buffered saline and weighed. The trout ventricle is composed of two distinct myocardial layers, a highly trabeculated inner spongy layer which makes up the majority of the organ (>80% in adult fish) and a thinner outer compact layer (Farrell and Jones, 1992; Pieperhoff et al., 2009). The apex of each ventricle was removed, the spongy tissue “scooped out” of the compact and stored at −80°C for quantitative real-time PCR (RT-qPCR). The remainder of the ventricle was bisected down the sagittal plane with one half snap frozen in OCT (Thermo Fisher Scientific, Waltham, MA, USA) by immersion in liquid nitrogen cooled 2-methylbutane (Sigma-Aldrich, St. Louis, MO, USA) and stored at −80°C. The other half was fixed in 10% neutral buffered formalin solution (Sigma-Aldrich, St. Louis, MO, USA) and embedded in paraffin wax so that sections would be cut in the transverse/axial plane.

### Histology

**Figure 1:**
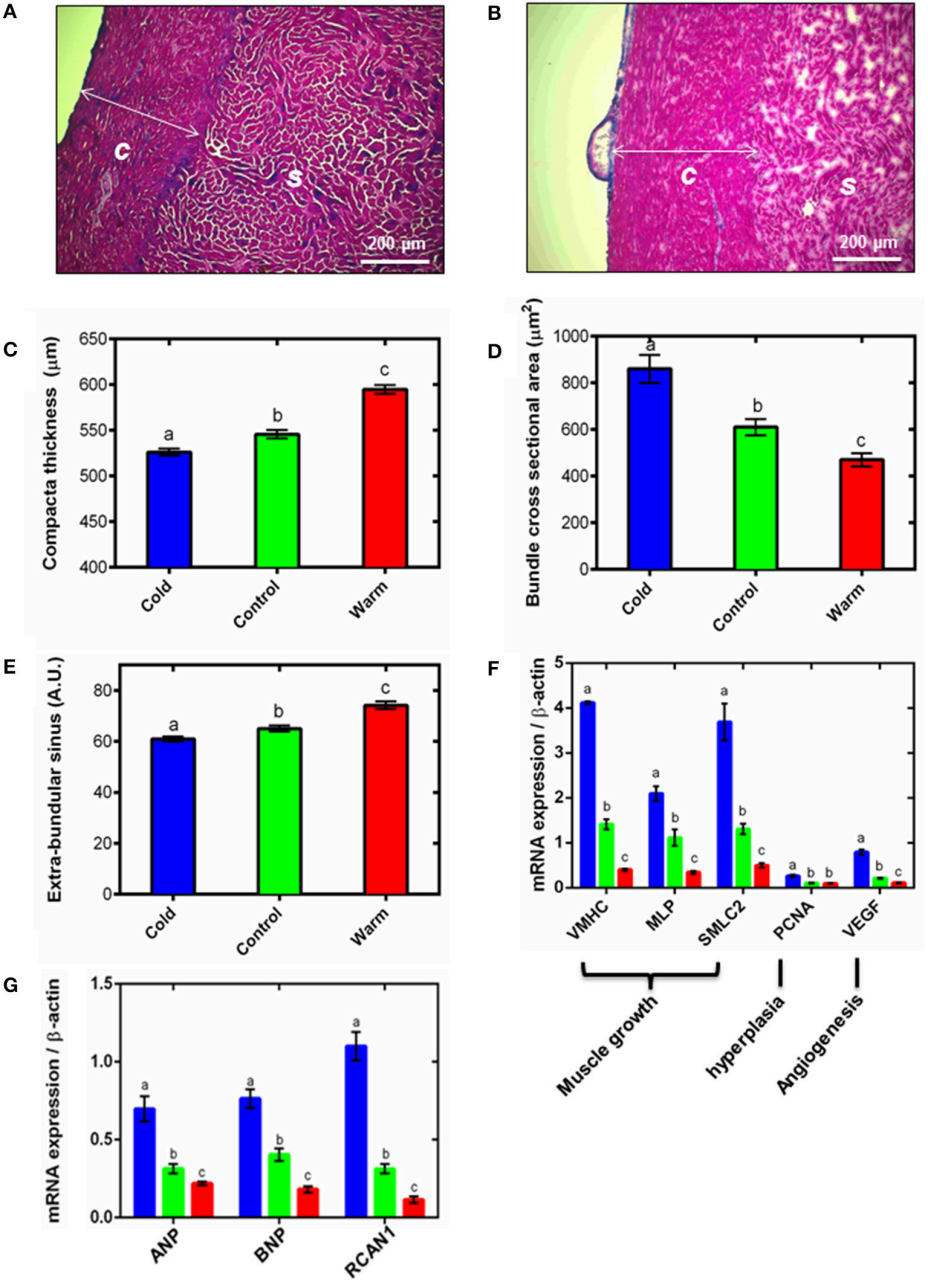
**Ventricular remodeling**. Representative Masson's trichrome stained cryo-sections for **(A)** cold (5°C) and **(B)** warm (18°C) acclimated rainbow trout show remodeling of the compact (*c*) and spongy (*s*) myocardium. Quantification of **(C)** compact layer thickness, **(D)** spongy myocyte bundle cross sectional area and **(E)** spongy layer extra-bundular sinus show remodeling with temperature acclimation. These differences are supported by **(F)** mRNA expression of gene markers of muscle growth (VMHC, MLP and SMLC2), hyperplasia (PCNA) and angiogenesis (VEGF) in the spongy myocardium. **(G)** mRNA expression of hypertrophic gene markers (ANP and BNP) and the pro-hypertrophic NFAT signaling pathway (RCAN1) in the spongy myocardium with cold (5°C; blue), control (10°C; green) and warm (18°C; red) acclimation (*n* = 7 fish for each acclimation group; 3 replicates for each animal were averaged for both histology and qPCR). Values presented are mean ± S. E. Significance was assessed by GLM with Holm-Sidak *post-hoc* test. Significance between groups is shown by dissimilar letters (*P* < 0.05).

All sections were cut to include both the compact and spongy layer so that differential remodeling between the ventricular layers could be evaluated histologically. Frozen tissue was sectioned at 10 μm (Leica CM3050S cryostat, Leica, Wetzlar, Germany), mounted onto glass slides (Super frost plus, Thermo Fisher Scientific, Waltham, MA, USA) and stained using Masson's trichrome (see Klaiman et al., 2011, for details). Previously, work on trout spongy tissue showed that cross-sections of single ventricular myocytes were not visible with Masson's trichrome staining. Rather, the visible structures were bundles of myocytes comprised of ~10–15 individual cells (see Klaiman et al., 2011). The myocyte bundle cross-sectional area can, therefore, be used as a proxy for myocyte cross-sectional area. Compact thickness, the cross-sectional area of myocyte bundles and extra-bundular sinus in the spongy layer were quantified using ImageJ software (Schneider et al., 2012). Masson's trichrome stains amorphous collagen bluish/purple (Figures 1A,B) and this was semi-quantified using ImageJ and the “threshold colour” plugin in binary mode (Klaiman et al., 2011). For morphometric analysis of compact layer thickness, myocyte bundle cross-sectional area and extra-bundular sinus eight sections were analyzed per individual fish. For compact layer thickness, 20 measurements were taken per image. For measurement of cross-sectional area of myocyte bundle area, on each tissue section three separate image montages were taken along transects across the full diameter of the cross section. In each image trabeculations were chosen for measurement only if they were in the transverse plane, i.e., the image showed a cross-section of the trabeculations making it circular in appearance. For extra-bundular sinus the non-tissue area of each image was measured.

Fibrillar collagen and elastin content were analyzed semi-quantitatively following Graham et al. (2011). Briefly, formalin-fixed tissue samples were processed, embedded in paraffin wax, sectioned at 5 μm (Leica RM2255 microtome, Leica, Wetzlar, Germany) and mounted onto glass slides. Serial sections from each sample were stained with picro-sirus red for collagen (Junqueira et al., 1979) and Miller's elastic stain for elastin (Miller, 1971). Picro-sirus red images were quantified using polarized light microscopy and Miller's elastic images were quantified using bright-field microscopy. Mean fibrillar collagen and elastin contents were expressed as a percentage of total tissue cross sectional area, excluding the epicardial surface, determined using ImageJ. Three tissue sections were considered for each individual to ensure consistency in measurements. On each tissue section three separate image montages were taken along transects across the full diameter of the cross section. All histological analysis was conducted blind to the acclimation group and in all cases these tissue sections were taken from the central 50% of the ventricle.

### Quantitative real-time PCR

As previous work has shown that cold-induced hypertrophy in fish occurs primarily in the spongy layer of the heart (Klaiman et al., 2011) and the compact layer was thin (~550 μm; making up ~17% of the ventricular area in the hearts used in this study; Poupa et al., 1974) all qRT-PCR was performed on spongy myocardial tissue only. Transcript abundance of genes associated with muscle growth (ventricular myosin heavy chain; VMHC, muscle LIM protein; MLP, and small myosin light chain 2; SMLC2), hyperplasia (proliferating cell nuclear antigen; PCNA), angiogenesis (vascular endothelial growth factor; VEGF), collagen I (Col1a1, Col1a2, and Col1a3), connective tissue regulators (MMP2, MMP9, MMP13, and TIMP2), stretch and heart failure (ANP and BNP) and pro-hypertrophic nuclear factor of activating T (NFAT) signaling mediator (regulator of calcineurin; RCAN1) were quantified in the ventricles of fish from cold, control and warm acclimated groups (*n* = 7 ventricles for each temperature). RNA was extracted from 5 mg of spongy tissue (RNeasyMicrokit, Qiagen, Venlo, NL) and amount and quality was determined (NanoDrop ND-1000, NanoDrop, Wilmington, DE, USA). An RNA concentration of 200 ± 50 ng μl^−1^ was used to make cDNA with SuperScript III First Strand Synthesis System (Invitrogen, Carlsbad, CA, USA). SYBR Green I pre-mixed chemo-technology was used for qPCR. qPCR was carried out in a 7900 HT sequence detection system (Applied Biosystems, Carlsbad, CA, USA) and cycle threshold (Ct) values generated by the qPCR machine were multiplied by primer efficiency (Pe) to determine gene expression levels (expression (e) = 1/(Ct ^*^ Pe). All primers were taken from published work (e.g., Johansen et al., 2011) or were designed using Primer 3 from mRNA sequences available on PUBMED. Specific marker genes and primers are in Table 2. All expression levels were normalized to housekeeping gene β-actin to determine absolute expression levels for comparison at each acclimation temperature. We tested three housekeeping genes; β-actin, GAPDH and DNAJ1, and β-actin had most stable expression in relation to temperature acclimation, as found previously (e.g., Johansen et al., 2011).

**Table 2:** **The specific marker genes with primers used for quantitative real-time PCR**.

**Gene**	**Primer pair**	**GenBank accession number**	**Function/marker**
***VMHC***	5′ – TGCTGATGCAATCAAAGGAA – 3′ 3′ – GGAACTTGCCCAGATGGTT – 3′	AY009126.1	Cardiomyocyte hypertrophy
***MLP***	5′ – AGTTCGGGGACTCGGATAAG – 3′ 3′ – CGCCATCTTTCTCTGTCTGG – 5′	NC007118.6 (*Danio rerio*)	Cardiomyocyte hypertrophy
***SMLC2***	5′ – GACAAGTTCA – 3′ 3′ – GGTTCTTGTAGTCC – 3′	NM001124678.1	Cardiomyocyte hypertrophy
***VEGF***	5′ – AGTGTGTCCCCACGGAAA – 3′ 3′ –TGCTTTAACTTCTGGCTTTGG – 5′	AJ717301.1	Angiogenesis
***PCNA***	5′ – AGCAATGTGGACAAGGAGGA – 3′ 3′ – GGGCTATCTTGTACTCCACCA′	EZ763721.1	Cardiomyocyte hyperplasia
***Col1a1***	5′ – GCTTTTGGCAAGAGGACAAG – 3′ 5′ – GCAGATAACTTCGTCGCACA – 3′	NM001124177.1	Fibrosis
***Col1a2***	5′ – GGCTGATCGGCTCTGTACTC – 3′ 3′ – TGGCTCTGCTGGTATCACTG – 3′	NM001124207.1	Fibrosis
***Col1a3***	5′ – CCCTGCTTTTTATGGTTGGA – 3′ 3′ – GCAGGGTTCTGGTTTCCATA – 5′	NM001124206.1	Fibrosis
***MMP2***	5′ – TGTATTGGGCAACATCAGGA – 3′ 3′ – CCCAGGAGACGATAGTCCAA – 5′	NC007118.6 (*Danio rerio*)	Inhibit fibrosis
***MMP9***	5′ – GGTCCAGTTTTCGTCATCGT – 3′ 3′ – AGACATGGGAGCCTCTCTGA – 5′	NM001124370.1	Inhibit fibrosis
***MMP13***	5′ – TCTGATGTGGTTTGCTGCTC – 3′ 3′ – CAGATAAGCCCGACCCTACA – 5′	NC007121.6 (*Danio rerio*)	Inhibit fibrosis
***TIMP2***	5′ – CAGGCCATCCACCTACTGTT – 3′ 3′ – TGTTGCTCTCTTGCATACGG – 5′	NC007123.6 (*Danio rerio*)	Inhibit MMPs
***ANP***	5′ – CCACAGAGGCTCTCAGACG – 3′ 3′ – ATGCGGTCCATCCTAGATC – 5′	NM001124211.1	Stretch/heart failure
***BNP***	5′ – TGGCCTTGTTCTCCTGTTCT – 3′ 3′ – GGAGACTCGCTCAACCTCAC – 5′	NM001124226.1	Stretch/heart failure
***RCAN1***	5′ – AGTTTCCGGCGTGTGAGA – 3′ 3′ – GGGGACTGCCTATGAGGAC – 5′	BC076439.1 (*Danio rerio*)	NFAT-activity/cardiomyocyte hypertrophic signaling
**β-*actin***	5′ – AGAGCTACGAGCTGCCTGAC – 3′ 3′ – GTGTTGGCGTACAGGTCCTT – 5′	NM001124235.1	Control/housekeeping

### *Ex vivo* passive pressure-volume curves

The intact isolated heart was placed into an organ bath containing Ringers solution [(in mM) 150 NaCl, 5.4 KCl, 2.0 CaCl_2_, 1.5 MgSO_4_, 0.4 NaH_2_PO_4_, 10 HEPES, 10 Glucose at a pH of pH 7.7 with NaOH at room temperature] at 10 ± 1°C to which 20 mM BDM (2, 3 butanedione monoxime) was added to prevent active cross-bridge cycling. Pressure-volume curves from ventricles from each acclimation group were generated at a common temperature, of 10 ± 1°C, to isolate the effects of chronic remodeling on myocardial stiffness from the acute effects of temperature. A cannula was fed through the atrium into the ventricular lumen and secured at the atrial-ventricular junction, using 0–0 silk thread (Harvard Apparatus, Holliston, MA, USA). An atraumatic clamp was placed at the bulbus-ventricular junction making the ventricle a sealed chamber with the cannula inside. The cannula was connected to a syringe pump (INFORS AG, Bottmingen, CHE), in series with a pressure transducer, containing 10 ± 1°C Ringer solution with BDM and a small amount of blue food coloring (Silverspoon, London, UK). The pressure transducer was calibrated daily against a static water column and recorded at 1000 Hz (Chart5, PowerLab, ADI Instruments, Dunedin, New Zealand). Ringer solution with BDM was pumped into the ventricle at 0.05 ml min^−1^ until maximum volume was achieved, determined by visual leak of the saline containing blue dye and a drop in the pressure trace.

### Atomic force microscopy (AFM)

Frozen ventricular tissue was sectioned at 5 μm (Leica CM3050S cryostat, Leica, Wetzlar, Germany) and mounted onto calcium fluoride (CaF_2_) slides. Excess OCT was removed with distilled water and the slides were left to dry for ~12 h. This methodology is consistent with previous work (Kemp et al., 2012; Wallace et al., 2012) which describes how tissue sections are best preserved dehydrated, with rehydration performed when nanomechanical measurements are required. Micro-indentation was carried out using a Bioscope Catalyst AFM (Bruker, Coventry, UK) mounted onto an Eclipse T1 inverted optical microscope (Nikon, Kingston, UK) fitted with a spherically tipped cantilever (nominal radius and spring constant of 1 μm and 3 Nm^−1^ respectively, Windsor Scientific Ltd., Slough, UK) running Nanoscope Software v8.15 (Bruker, Coventry, UK). The local reduced modulus was determined for each of 400 points in a 50 × 50 μm region, indented at a frequency of 1 Hz with lateral spacing of 2.5 μm. The extend curve was used in conjunction with a contact point based model to calculate the reduced modulus for each indentation (Crick and Yin, 2007). For each biological sample, 400 force curves were collected at three distinct 50 μm^2^ regions. Once all 400 force curves had been generated, quality control was applied whereby any force values falling more than two standard deviations away from the mean value were discarded in order to account for failed indents. Data loss at this stage was less than 10% (data not shown).

### Statistical analysis

Chamber filling volume was calculated from filling time by the equation:

$$volume(ml)=time(\mu s)\times \frac{0.05}{60}\times 1000$$

The effect of temperature acclimation on the pressure-volume relationship was assessed by a general linear model (GLM) with pressure as the dependent variable, volume and acclimation group as fixed factors and body mass as the covariate, with a Tukey *post-hoc* test for differences between groups using R (R Core Team, 2013). The calculations were performed on all data below 2 kPa, which approximates the maximum physiological pressures experienced by this species (Forster and Farrell, 1994). Differences in compact myocardial thickness, myocyte bundle cross sectional area, extra-bundular sinus, collagen deposition and transcript abundance were assessed by GLM with Holm-Sidak *post-hoc* test for differences between groups using SigmaPlot 11.0 (SYSTAT Statistics, San Jose, CA, USA). *Post-hoc* analyses of AFM force curves were performed using Nanoscope Analysis v1.40 (Bruker, Coventry, UK), whereby a baseline correction was applied to each curve before a force fit was applied using a Herzian (spherical) model and a maximum force fit of 70%. For all analyses significance was considered to be *P* < 0.05, except for atomic force curves where significance was considered at *P* < 0.005. Values are presented as mean ± S. E. throughout except for atomic force curves where values are mean ± S. D. Statistical details are provided in the figure legends.

## Results

### Thermal remodeling of ventricular muscle

Thermal acclimation caused differential and opposite remodeling of the two myocardial layers within the fish ventricle (Figures 1A–C) which is in line with previous findings (Gamperl and Farrell, 2004; Klaiman et al., 2011, 2014). Compared to controls, the compact layer (Figures 1A,B) was 4% thinner after cooling and 9% thicker after warming (*P* < 0.05; Figure 1C). However, there was no difference in total ventricular mass or ventricular mass relative to body mass (RVM) between the three temperature acclimation groups (Table 1). Changes in compact layer thickness with no change in overall ventricular mass indicates compensative remodeling of the spongy myocardial layer, which was detected as an increase in the cross-sectional area of the myocyte bundles that make up the spongy trabeculae (Klaiman et al., 2011). Cross-sectional area of cold-acclimated myocyte bundles was 83% greater than warm-acclimated bundles (*P* < 0.05; Figure 1D) and correlated with a reduced sinus space between bundles (*P* < 0.05; Figure 1E). Cold-induced spongy hypertrophy and warm-induced spongy atrophy were supported by changes in mRNA expression of muscle-specific growth genes (Figure 1F) with VMHC 10.3-fold higher, MLP 6.1-fold higher, and SMLC2 7.4-fold higher in cold- compared with warm-acclimated spongy myocardium (*P* < 0.05, Figure 1F). In addition, PCNA, a marker for hyperplasia, was 2.6-fold higher, in the cold- compared with the warm-acclimated spongy myocardium (Figure 1F). VEGF, a marker for angiogenesis, was also higher (7.3-fold) in cold compared with warm myocardium.

Spongy layer and myocyte bundle hypertrophy requires increased protein synthesis, which can occur as a result of hypertrophic signaling cascades. In mammals, RCAN1 is a target gene of the NFAT signaling pathway and promotes pathological hypertrophic growth via the fetal gene program, which has also recently been shown in fish (Wilkins et al., 2004; Bernardo et al., 2010; Johansen et al., 2011; Shih et al., 2015). We found RCAN1 mRNA expression was 9.5-fold higher in cold- compared with warm-acclimated spongy myocardium (*P* < 0.05; Figure 1G). ANP and BNP are released by cardiomyocytes in response to myocardial stretch induced by pressure and volume overload (Kinnunen et al., 1993). mRNA expression of ANP was 3.2-fold higher and BNP was 4.2-fold higher in cold- compared with warm-acclimated animals (*P* < 0.05; Figure 1G).

### Thermal remodeling of connective tissue

**Figure 2:**
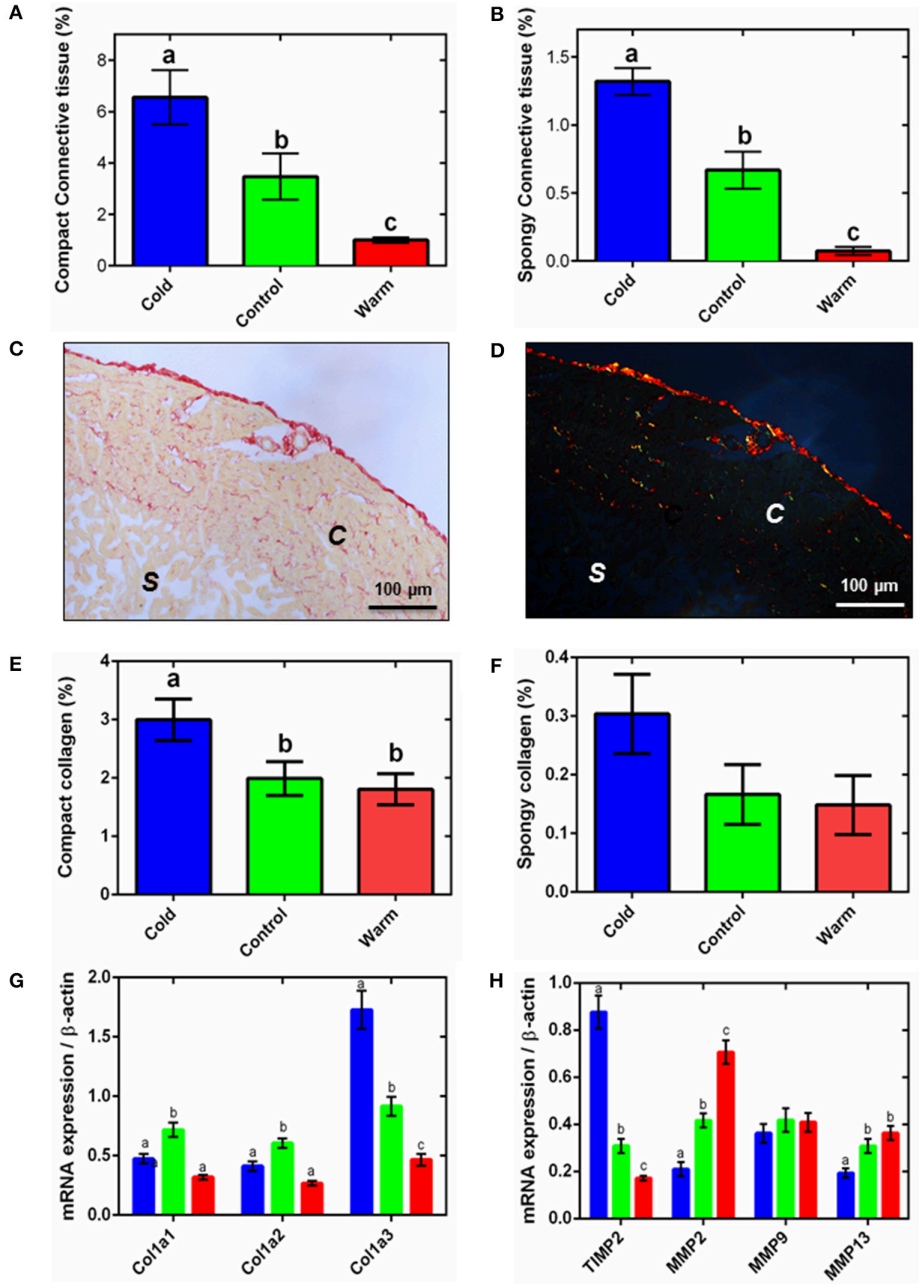
**Ventricular connective tissue remodeling**. Semi-quantification of amorphous collagen from Masson's trichrome histology for compact tissue **(A)** and spongy tissue **(B)**. Representative **(C)** bright-field and **(D)** polarized micrographs of sections of ventricular tissue stained with picro-sirus red, showing the spongy (*s*) and compact (*c*) myocardium. Semi-quantitative analysis of picro-sirus red stained sections **(E)** compact and **(F)** the spongy myocardium. In **(A,B,E,F)**, collagen content is expressed as a percentage of total tissue for either the compact or spongy layer. The corresponding mRNA expression of **(G)** collagen genes and **(H)** collagen regulatory genes (TIMP2, up-regulation; MMP2, MMP9, and MMP13, down-regulation) in the spongy myocardium of cold (5°C; blue), control (10°C; green), and warm (18°C; red) acclimated rainbow trout (*n* = 7 fish for each acclimation group; 3 replicates for each animal were averaged for both histology and qPCR). Values presented are mean ± S. E. Significance was assessed by GLM with a Tukey, or Holm-Sidak for multiple comparisons, *post-hoc* test. Significance between groups is shown by dissimilar letters (*P* < 0.05).

Myocyte remodeling is associated with remodeling of the extracellular matrix (ECM) in both fish and mammals (Chapman et al., 1990; Klaiman et al., 2011). Similar to our earlier work (Klaiman et al., 2011) we found amorphous collagen deposition was greater in both compact and spongy layers with cold acclimation (*P* < 0.05; Figures 2A,B). We then extended this finding using picro-sirus red to determine fibrillar collagen. Figure 2C shows a representative bright-field ventricular section stained with picro-sirus red and Figure 2D shows the same section visualized under plane polarized light. The polarized light images were used to quantify collagen as a percentage of either compact or spongy tissue area. In agreement with results from Masson's trichrome staining (Figures 2A,B), compact myocardial collagen content was 1.7-fold higher in cold-acclimated than control and warm-acclimated animals (*P* < 0.05; Figure 2E). Despite a trend, we found no statistical differences between acclimation groups for fibrillar collagen in the spongy layer (Figure 2F). We did not detect elastin in the fish ventricular myocardium except in coronary vessels (not shown).

The changes in collagen content were supported by differential expression of the collagen I gene, Col1a3. In mammals, collagen I accounts for ~80% of total collagen in the myocardium and is the main collagen in cardiac fibrosis (Medugorac, 1982). Mammalian collagen I is composed of type 1 (α1) and type 2 (α2) alpha-helical chains; fish also have an additional type 3 (α3) chain (Saito et al., 2001). We found expression of this Col1a3 gene was 1.4-fold higher in the cold- compared with the warm-acclimated ventricles (*P* < 0.05; Figure 2G). Expression of the Col1a1 and Col1a2 mRNA was lower in both cold and warm fish compared with controls, suggesting temperature-independent remodeling is also occurring. Total collagen content is a balance between deposition and degradation by matrix metalloproteinases (MMPs) (Nagase et al., 2006). MMP2 and MMP13 were 3.4- and 1.9-fold higher, respectively, in the warm- compared to cold-acclimated ventricle (*P* < 0.05; Figure 2H). MMP9 did not differ between groups. MMP activity is regulated by tissue inhibitors of MMPs (TIMPs). TIMP activity inhibits collagen degradation by MMPs and is, thus, associated with increased collagen deposition. Expression of the TIMP2 gene was 5.1-fold higher in the cold- compared with the warm-acclimated ventricle (*P* < 0.05; Figure 2H).

### Thermal remodeling of *ex vivo* chamber compliance

**Figure 3:**
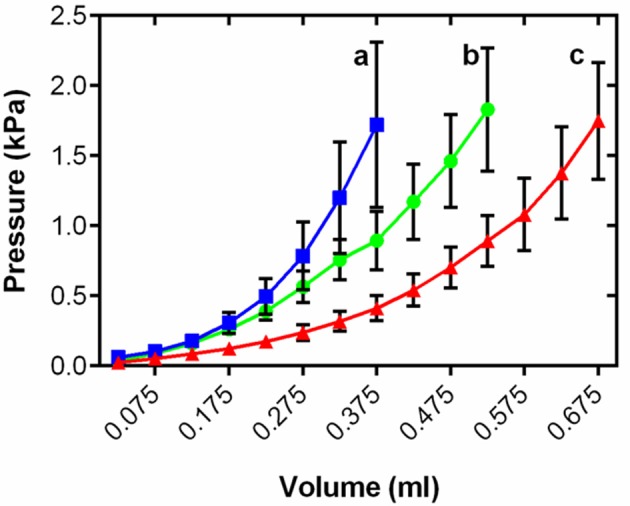
**Mean ventricular passive pressure-volume relationships within the physiological relevant pressure range of <2 kPa for cold (5°C; blue), control (10°C; green), and warm (18°C; red) acclimated rainbow trout (*n* = 8)**. Values are mean ± S. E., curves show all mean data for *n* > 3. Pressure has been standardized to start at 0 kPa for graphical representation. Significance of changes in compliance with temperature acclimation was assessed by GLM with volume as the dependent variable, treatment and pressure as the fixed factors and chamber mass as the covariate (*P* < 0.05) and is shown by dissimilar letters.

Changes in myocardial thickness and fibrosis are known to influence chamber compliance in mammals (Bing et al., 1971). To assess the functional effects of cardiac remodeling on the passive properties of the thermally acclimated fish ventricle we generated *ex vivo* passive filling curves from freshly isolated intact ventricles treated with BDM at a common test temperature of 10°C. Figure 3 shows mean data for each temperature within the range of physiologically relevant filling pressures experienced by rainbow trout *in vivo* (Forster and Farrell, 1994). Thermal acclimation altered the pressure-volume relationship during filling [*R*^2^ = 0.60, *F*_(2, 15, 575)_ = 1447.0, *P* < 0.001] revealing greater stiffness in the cold and greater compliance in the warm compared to controls (t ratio = 54.2; Figure 3).

### Micromechanical ventricular stiffness

**Figure 4:**
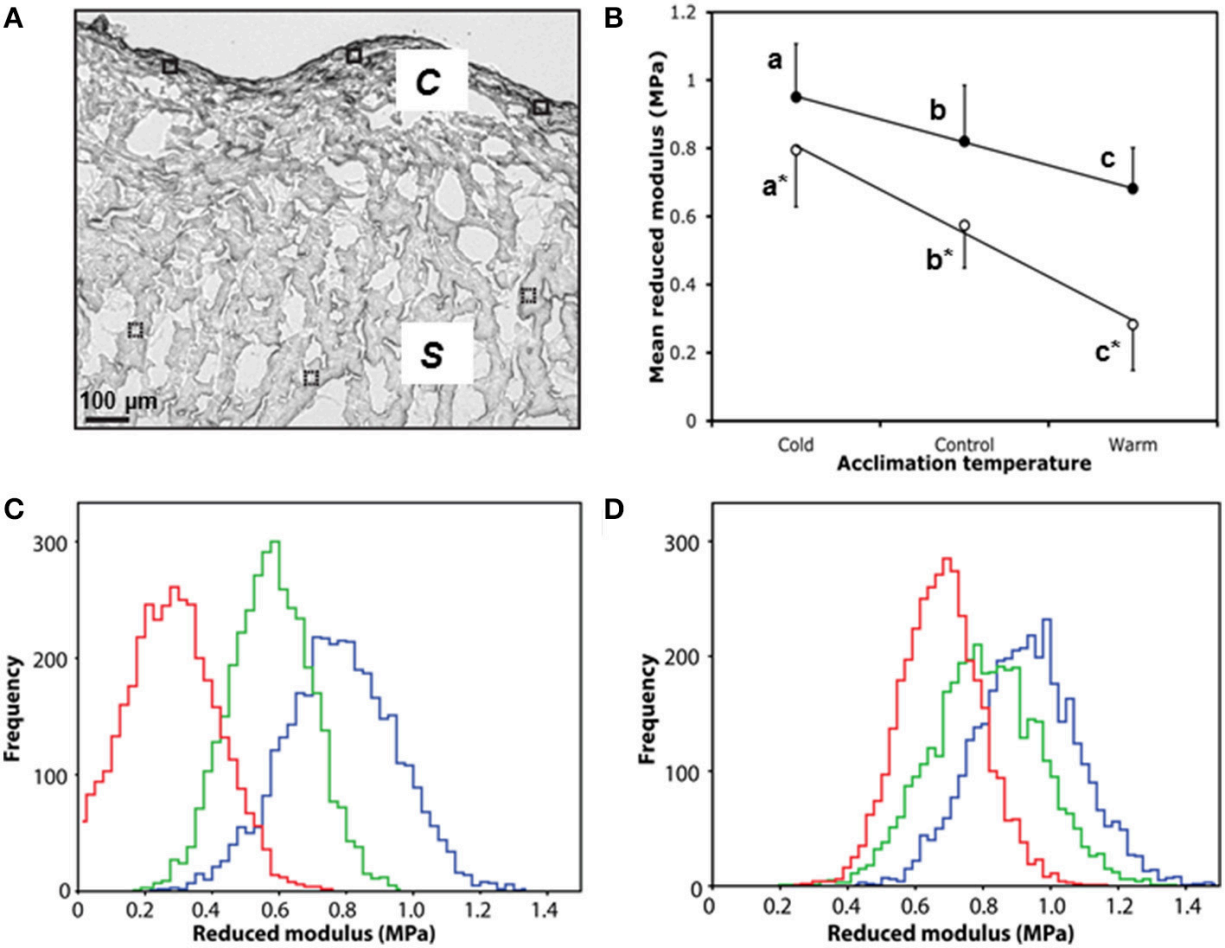
**Micromechanical testing of compact and spongy myocardium by AFM indentation**. **(A)** White light microscope image of control ventricle showing six 50 × 50 μM scan areas used for AFM indentation, closed for compact (c), and dashed for spongy (*s*). **(B)** Mean reduced modulus (E_r_) of spongy (open circles) and compact (closed circles) myocardium by acclimation temperature. **(C)** Accumulative frequency curves of each individual E_r_ from the spongy myocardium and **(D)** the corresponding frequency curves for the compact myocardium with cold (5°C; blue), control (10°C; green) and warm (18°C; red). Values presented are mean ± S. D. Significance was assessed by GLM and is shown between groups by dissimilar letters and by ^*^ between tissue types (*P* < 0.005).

Collagen is an important mediator of tissue tensile strength and stiffness, and is arranged into networks that support cardiomyocytes. As alterations in collagen content are known to affect cardiac micromechanical properties (Fomovsky et al., 2010), we used AFM indentation of ventricular cryo-sections to assess whether the temperature-dependent collagen remodeling was associated with a change in local tissue stiffness (Figure 4A). Cold-acclimated ventricular tissue was stiffer than warm-acclimated tissue (*P* < 0.005; Figure 4B). Mean reduced modulus (E_r_; and hence localized tissue stiffness) was strongly and significantly correlated with temperature in both spongy (*R*^2^ = 0.99, *P* < 0.0001) and compact myocardium (*R*^2^ = 1.00, *P* < 0.0001; Figure 4B), with the effects of temperature being more pronounced in spongy tissue compared with compact tissue. Furthermore, the modulus frequency distribution in both tissue types (Figures 4C,D) suggests that mechanical remodeling following temperature acclimation is due to homogenous structural and/or compositional remodeling of the whole tissue, rather than isolated or specific regions of the tissue.

## Discussion

Seasonal changes in temperature trigger remodeling of the fish heart (Farrell et al., 1988; Graham and Farrell, 1989; Pelouch and Vornanen, 1996; Aho and Vornanen, 1999, 2001; Gamperl and Farrell, 2004; Vornanen et al., 2005b; Hassinen et al., 2008; Korajoki and Vornanen, 2009, 2012; Klaiman et al., 2011, 2014; Johnson et al., 2014). Here, we focused on the passive properties of the rainbow trout ventricle across multiple levels of biological organization following chronic warming and chronic cooling. Our principle and novel findings are: (1) Cold acclimation increases fibrillar collagen deposition, the expression collagen promoting genes and markers for mammalian pathological remodeling including those associated with the fetal gene program (RCAN1, ANP, BNP). Each of these observations changed in the opposite direction following chronic warming. (2) Cold-induced fibrosis and hypertrophy were associated with increased passive stiffness of the whole ventricle and increased micromechanical stiffness in tissue sections. Again, the opposite response occured with chronic warming. These findings provide support for our hypothesis that chronic cooling in the fish heart invokes a hypertrophic phenotype with increased cardiac stiffness, fibrosis and hypertrophic markers analogous to pathological hypertrophy in the mammalian heart. Importantly, we report a suppression of growth genes and hypertrophic markers following chronic warming. This is novel and provides a potential new investigative route for understanding processes that regulate regression of pathological hypertrophy. We thus propose the trout heart as a potential vertebrate model for investigating regression of fibrotic cardiac hypertrophy.

### Cold-induced hypertrophy in the spongy myocardium

The trabecular nature of the spongy myocardium in fish is important as it increases the surface area and reduces the distance for diffusion of gases and nutrients between the myocardium and the venous blood that transits the ventricular lumen. Fish have a single circulation; the heart sends deoxygenated blood to the gills to be oxygenated, which travels around the body before returning to the heart. Thus, the spongy layer of the fish heart receives oxygen-poor venous blood. The thickness of the spongy myocardium is, therefore, a compromise between minimizing the distance for oxygen diffusion and maximizing the cross-sectional area for tension development (Davie and Farrell, 1991). We have shown spongy layer cardiac hypertrophy following cold acclimation, and atrophy following warm acclimation, indirectly as reciprocal changes in myocyte bundle cross-sectional area in the trabeculated region of the heart and by changes in the expression of hypertrophic genes. Similar molecular responses have been observed in fish with stress-induced cardiac hypertrophy (Johansen et al., 2011) and chronic cooling (Vornanen et al., 2005a), but the reciprocal responses with warming have not, to our knowledge, been previously reported. It should be noted that we did not measure the cross-section area or volume of the single myocytes in our study, which is the base unit of hypertrophy. However, previous work in this species with similar temperatures (e.g., Vornanen, 1998; Clark and Rodnick, 1999; Vornanen et al., 2005a) do report single cell hypertrophy, associated with changes in temperature and cardiac load, and importantly correlate this with the changes in gene expression (VHMC, SMLC2, and MLP; Vornanen et al., 2005a) we show here. Our interpretation of cold-induced hypertrophy is in line with our previous work (Klaiman et al., 2011) and also a recent mathematical model of the spongy layer of the carp heart (*Cyprinus carpio*) which shows increasing trabecular tissue volume increases stroke volume, stroke work and ejection fraction (Kochová et al., 2015).

Our novel finding of increased mRNA expression of PCNA suggests hyperplasia may also contribute to the growth of the myocyte bundles in the spongy layer during cold acclimation. Cardiac hyperplasia has been previously reported during normal growth of rainbow trout (Farrell et al., 1988) and in exercise trained Atlantic salmon (Castro et al., 2013). The fish myocardium is also known for its regenerative ability, which requires cell proliferation and hyperplasia (Sun et al., 2009). We cannot, therefore, exclude hyperplasia of myocytes or other cell types, such as fibroblasts, in response to chronic cooling. However, the changes in gene expression of hypertrophic-related genes (VMHC, MLP and SMLC2) were between 6.1-fold and 10.3-fold higher in the cold compared with the warm, whereas those of the hyperplasia marker (PCNA) changed by 2.6-fold.

Hypertrophic growth follows activation of downstream hypertrophic signaling cascades. Mitogen-activated protein kinases (MAPKs) and the calcineurin-NFAT pathway are central to pathological hypertrophic growth in mammals (Wilkins et al., 2004; Bernardo et al., 2010). RCAN1 can increase calcineurin-NFAT signaling and enhance hypertrophic growth of myocytes (Liu et al., 2009). We found an up-regulation of RCAN1 in the cold- and a down regulation in warm-acclimated trout ventricle suggesting components of the pathological hypertrophic signaling cascade in mammals are also present in fish. The calcineurin-NFAT signaling system is largely unexplored in the adult fish heart, although RCAN1 has been linked to the hypertrophic response to stress in rainbow trout (Johansen et al., 2011). Calcineurin-NFAT signaling has also been shown to be important in heart development of zebrafish, indicating a physiological role under non-hypertrophic conditions (Armstrong et al., 2003). However, it is important to note that, although fetal and adult cardiac growth pathways in zebrafish show a high level of conservation with those of mammals (Shih et al., 2015), the adult phenotype of the fish heart likely more closely resembles a mammalian neonate than a mammalian adult (Tibbits et al., 2002) as some genes characteristic of the mammalian fetal gene program, such as ANP, are intrinsically expressed in the adult fish heart (Sun et al., 2009; Jensen et al., 2012).

We did not measure gene expression or myocyte size in the compact myocardial layer, which had inverse thermal remodeling to the spongy layer. This limits interpretation of transmural remodeling across layers in our study. Unlike the spongy layer, which receives venous blood, the compact layer of the fish ventricle has a coronary blood supply from the gill (Farrell and Jones, 1992). The cold-induced atrophy of the compact layer may reflect reduced reliance on coronary circulation due to an increased oxygen carrying capacity of water and the blood, and a decreased oxygen demand from cardiac muscle at cold temperatures, as suggested by Farrell and Clutterham (2003). This idea is supported by recent work showing increased VEGF expression in the compact layer of warm-acclimated Atlantic salmon (Jørgensen et al., 2014). The reduction in compact thickness after chronic cold exposure could also increase cardiac compliance following cold-induced stiffening (Johnson et al., 2014; Klaiman et al., 2014).

### Connective tissue remodeling

We found amorphous collagen increased in both myocardial layers following chronic cold which confirms our earlier findings (Klaiman et al., 2011). We extended this work by showing that fibrillar collagen is also increased following cooling. Collagen is the primary support protein in the ECM and is associated with pathological remodeling in mammals. Accordingly, we report an increase in the fish-specific Col1a3 gene expression in the spongy layer from the cold-acclimated ventricle. Cold-induced fibrosis is thought to maintain mechanical cardiac performance under elevated hemodynamic stress of pumping cold, highly viscous blood (Cerra et al., 2004). The pro-collagen regulatory enzyme TIMP2 increased with cold acclimation and the anti-collagen regulatory MMP genes decreased with cold acclimation supporting this remodeling. In fish, MMP13 catalyses the hydrolysis of collagen, degrading it to gelatin (Hillegass et al., 2007), and MMP2 and MMP9 digest the gelatin into removable waste products (Kubota et al., 2003). Although the picro-sirus red histological analysis did not show a statistically resolvable increase in spongy layer collagen, we show up-regulation of collagen regulatory genes which may pre-empt the fibrotic response. Interestingly, the reverse has been shown with cooling zebrafish (Johnson et al., 2014) and Atlantic salmon (Jørgensen et al., 2014) where a reduction in ventricular collagen and collagen-related genes expression has been documented. These differences may be species-specific; however, varying myocardial responses to thermal acclimation in fish are commonly reported (Sephton and Driedzic, 1995; Johnson et al., 2014; Klaiman et al., 2014). Sex, maturation and circannual rhythms are thought to influence the degree of overall hypertrophy (Clark and Rodnick, 1999; Gamperl and Farrell, 2004; Klaiman et al., 2011, 2014). In our study, it is likely that the winter photoperiod, in addition to 8 week chronic cold, played an important role in the onset of spongy layer and myocyte bundle hypertrophy.

### Ventricular compliance

Temperature acclimation significantly altered passive pressure-volume relationships in the ventricle. Cold-acclimated tissue was stiffer and warm-acclimated tissue was more compliant than control tissue when all were tested at a common temperature. Although diastolic filling curves have previously been generated for fish hearts (Forster and Farrell, 1994; Mendonça et al., 2007), this is the first study where they have been used to probe remodeling. Klaiman et al. (2014) used a Langendorff preparation to generate working pressure-volume loops in the trout ventricle and found that temperature acclimation affected the systolic, but not diastolic phase of the cardiac cycle. However, this study did not find cold-induced fibrosis which could explain stasis of diastolic stiffness. Our histological and gene expression data suggest that although collagen deposition/regression is a strong driver in the changes in functional compliance following thermal acclimation, other factors must also be at play. It is likely that other temperature dependent components of the myocardium are affected, such as the actin cytoskeleton (Qiu et al., 2010) which could alter intrinsic stiffness of the cardiomyocytes. We have previously shown that titin isoform expression in trout ventricle underlies the compliance of the fish myocyte (Patrick et al., 2009). How titin changes with thermal remodeling is currently unknown, but could be crucial in understanding tissue and organ level compliance.

The strong negative correlation between temperature and micromechanical stiffness in both the spongy and compact myocardium suggests that the changes in tissue ultrastructure and matrix organization are exerting a functional effect on the mechanical competency of the myocardium. Previous *in situ* studies show increased stroke volume and *in vitro* studies have shown increased Ca^2+^ sensitivity of force generation following cold acclimation in salmonid hearts (Graham and Farrell, 1989; Franklin and Davie, 1992). Our work on the passive properties builds from these studies on the active properties by suggesting that the temperature-mediated changes in cardiac force and/or cardiac output must compensate for the altered micromechanical stiffness. Remodeling of the passive components of the fish heart with cold results in a ventricle more capable of generating the forces required to maintain blood pressure at cold temperatures, whilst being protected from overstretch from pumping viscous blood. Clearly, the cold-induced fibrosis in the fish heart is not pathological and the fibrotic phenotype is more plastic in fish than in mammals.

### Relevance of thermal remodeling to mammalian hypertrophy

Here, we have compared our findings of cardiac remodeling in the fish ventricle to those in physiological and pathological remodeling of the mammalian ventricle. However, it should be noted that the architecture and development of the fish heart differs from the mammalian heart. The mature mammalian heart is almost entirely compact with integrated trabeculae (Weiford et al., 2004; Risebro et al., 2006), whereas, the trout ventricle is formed of two distinct layers; an outer wall of compact myocardium and an inner layer of trabecular myocardium (Pieperhoff et al., 2009). Throughout this study we have focused on the inner spongy layer for our comparison due to the cold-induced spongy layer hypertrophy. Future studies should investigate the effect of temperature acclimation on the thermal remodeling of the outer compact layer of the fish heart which may be more relevant to mammalian heart remodeling. However, although many of the genes and signaling pathways that regulate cardiac development and morphogenesis are shared between all vertebrates (Harvey, 2002; Luxán et al., 2013; Samsa et al., 2013) the compact layer of a fish heart is not directly analogous to that of adult mammals (Gupta and Poss, 2012).

### Perspectives

Hypertrophy and stiffening of the ventricle, with associated fibrosis, are characteristics of mammalian pathological remodeling. In fish, it is likely that reactive fibrosis occurs to provide support for increasing muscle mass and to prevent over stretching of myocytes under increased hemodynamic stress in the cold. This increased stiffness during cold acclimation is likely protective, despite fibrosis. We did not determine whether chronic cold induces remodeling beyond that necessary for accommodating the changes in blood viscosity. To understand whether temperature pushes the fish heart past the compensation/adaptation phase into the true pathology, future studies could combine approaches known to induce pathology in mammalian hearts (e.g., outflow constriction) in fish hearts.

Our finding of opposite cardiac remodeling in chronically warmed fish, compared with control fish and compared with chronically cooled fish is intriguing as it suggests fibrosis may oscillate seasonally, revealing a more dynamic nature than fibrosis associated with dysfunction in mammals. This finding builds upon previous work to suggest an opposite remodeling phenotype in fish following chronic warming compared to chronic cooling (e.g., Klaiman et al., 2011, 2014). However, to directly test whether the cardiac remodeling response is “reversible,” cardiac function would have to be assessed in the same animals following acclimation to both temperatures (i.e., cooling then warming or warming then cooling). Moreover, the collagen I gene that appeared most responsive to thermal remodeling in the current study is fish-specific (Col1a3). Collagen chains containing this domain have been shown to have greater susceptibility to heat denaturation and degradation by MMP13 than collagen chains without it (Saito et al., 2001), which may explain its malleability with chronic temperature change. If this collagen domain is driving the fish fibrotic response to cold, it may explain why a typically pathological response in mammals occurs transiently in fish.

## Author contributions

AK, AF, PG, and HS are responsible for the concept and design of the research. AK, AF, and JM performed experiments and analysed data in HS and MS laboratories. AK, AF, JM, MS, and HS interpreted the results of the experiments. AK, JM, and HS drafted the manuscript. AK, AF, JM, MS, PG, and HS revised and edited the manuscript. AK, AF, JM, MS, PG, and HS approve the final version of the manuscript submitted for publication.

## Funding

AK and AF were supported by studentships from the BBSRC. The Shiels lab is supported by the Leverhulme Trust (240613). MJS is funded by the Medical Research Council UK (grant G1001398).

### Conflict of interest statement

The authors declare that the research was conducted in the absence of any commercial or financial relationships that could be construed as a potential conflict of interest.

## Abbreviations

AFM: atomic force microscopy
ANP: atrial natriuretic peptide
BNP: brain natriuretic peptide
Col1a1: collagen I alpha 1
Col1a2: collagen I alpha 2
Col1a3: collagen I alpha 3
ECM: extracellular matrix
GLM: general linear model
MLP: muscle LIM protein
MMP2: matrix metalloproteinase 2
MMP9: matrix metalloproteinase 9
MMP13: matrix metalloproteinase 13
NFAT: nuclear factor of activating T
PNCA: proliferating cell nuclear antigen
RCAN1: regulator of calcineurin 1
RT-qPCR: real-time quantitative PCR
SMLC2: small myosin light chain 2
TIMP2: tissue inhibitor of metalloproteinase 2
VEGF: vascular endothelial growth factor
VMHC: ventricular myosin heavy chain.
